# Supplementary material for: SyNDI: synchronous network data integration framework
Source: BMC Bioinformatics. 2018 Nov 6;19:403. doi: 10.1186/s12859-018-2426-5 (PMC6219086; doi:10.1186/s12859-018-2426-5)
Supplement: Supplementary file 5 — Files of the example presented in Additional file 4. This additional file is a zip package that contains all files used in Additional file 4. Detailed descriptions of each of these files are presented in a README file included in this zip package. (GZ 30411 kb) [file 12859_2018_2426_MOESM5_ESM.gz › SyNDITools/Meme2Fimo/meme_4.9.0_4.tar.gz!/meme_4.9.0/doc/draw-mhmm.xml]

draw-mhmm


# draw-mhmm -- not currently supported

**Usage:** draw-mhmm [options] <*HMM file*>

**Description:**

> 'draw-mhmm' converts a given Meta-MEME hidden Markov model into
> a format suitable for input to the graphviz
> program from AT&T Research.

**Input:**

- <*HMM file*> - An HMM in Meta-MEME format (as
  produced by mhmm) must be provided. If the
  filename is given as '-', then 'mhmms' will attempt to read the HMM
  from standard input.

**Output:**

> The program writes to standard output a description of the HMM
> in graphviz format.

**Options:**

- --mintrans <*value*> - Only include in the graph
  edges whose transition probabilities exceed the given value.
- --consensus - Print consensus along with motif id.
- --verbosity 1|2|3|4|5 - Set the verbosity level of the output
  to stderr. The default level is 2.

**Bugs:** None known.

Author: William
Stafford Noble.
